# Glutamic Acid as Repeating Building Block for Bio-Based Films

**DOI:** 10.3390/polym12071613

**Published:** 2020-07-20

**Authors:** Mohammed Sabbah, Prospero Di Pierro, Francesco Ruffo, Chiara Schiraldi, Alberto Alfano, Marcella Cammarota, Raffaele Porta

**Affiliations:** 1 Department of Nutrition and Food Technology, An-Najah National University, P.O. Box 7, Nablus 44859, Palestine; m.sabbah@najah.edu; 2 Department of Chemical Sciences, University of Naples “Federico II”, Montesantangelo Campus, via Cintia 4, 80126 Naples, Italy; prospero.dipierro@unina.it (P.D.P.); francesco.ruffo@unina.it (F.R.); 3 Department of Experimental Medicine, Section of Biotechnology and Molecular Biology, University of Campania “Luigi Vanvitelli”, 80138 Naples, Italy; chiara.schiraldi@unicampania.it (C.S.); alfano84@libero.it (A.A.); marcella.cammarota@unicampania.it (M.C.)

**Keywords:** poly-γ-glutamic acid, bioplastics, bio-based films

## Abstract

Commercial inexpensive preparations of poly-γ-glutamic acid were used to obtain films made with a polypeptide constituted by a single repeating unit. The homopolymer was characterized by ^1^H-NMR spectroscopy and thermogravimetry, as well as by zeta potential and Z-average measurements. Manipulatable materials were obtained by casting film-forming solutions prepared at pH values between 3.0 and 4.0 and containing extensively dialyzed samples of the commercial product. The analysis of the mechanical properties highlighted a marked extensibility and plasticity of the films obtained without plasticizer, even though the addition of low amounts of glycerol (1–4%) was able to further increase these features. The characterization of poly-γ-glutamic acid molecular species, performed by membrane ultrafiltration and size-exclusion chromatography, coupled with triple-detection analysis of the obtained fractions, suggested that biopolymer chain length is responsible not only for its capacity to form film, but also for conferring to the films different features depending on the homopolymer molecular weight.

## 1. Introduction

The conversion of biodegradable raw material in bioplastic products represents one of the major objectives in the development of sustainable processes. In this context, the application of biomass constituted by polysaccharides and proteins to substitute fossil resources for the production of biodegradable/edible materials is a widely accepted strategy [1,2]. The last few decades witnessed the development of numerous biopolymers, which gave rise to ecofriendly materials, potential alternatives to the polymers that are derived from petroleum sources. However, their application has so far been restricted because they are mechanically weaker and tend to have higher water vapor permeability as compared to most synthetic polymers, in addition to their limited thermoplastic processability. Nevertheless, many efforts are being made to enhance the physical and barrier properties of hydrocolloidal films. The most promising attempts concern film formulation and preparation conditions, including plasticization, pH change, and nanoparticle addition, as well as chemical or enzymatic cross-linking. Different film-forming solutions (FFSs) have thus been investigated by blending various biomacromolecules under different experimental conditions and in the presence of a variety of plasticizing and/or structuring additives [3,4,5,6,7,8].

Traditional plastics are constituted by polymers comprising repeating units that determine the specific properties of the different plastics. The monomers more frequently present in plastics able to be shaped or molded include ethylene, propylene, styrene, formaldehyde, and vinyl chloride [9]. The repeat of the same unit in the polymer seems to represent a frequent requirement of the plastic materials in order to possess acceptable performance, as demonstrated not only by polyethylene, polypropylene, polystyrene, and polyvinyl chloride, but also by the biopolymers such as poly-lactic acid, starch, and cellulose derivatives, made by the repeat in the sequence of lactic acid and glucose monomeric units, respectively. Conversely, heteropolysaccharides and proteins are biopolymers containing similar but not identical repeating units. In particular, amino acids with different lateral chains characterize the sequence of a protein, determining its tridimensional structure and, consequently, its different behavior during drying and the properties of the deriving material.

**Figure 1:**
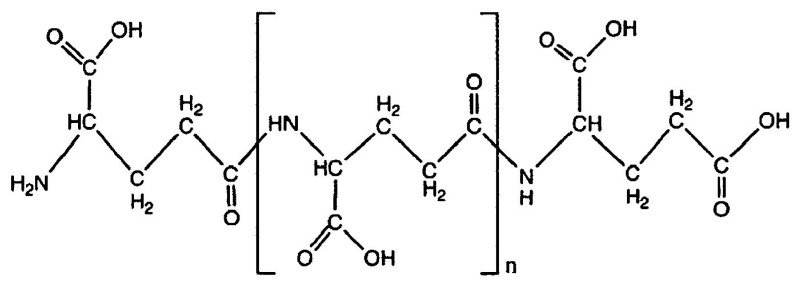
Structure of poly-γ-glutamic acid (PGA).

Little is known on the ability of polypeptides constituted by the same amino acid to give rise, under selective experimental conditions, to materials with plastic-like properties. Among these, poly-γ-glutamic acid (PGA) is a biodegradable/edible, naturally occurring poly-amino acid synthesized by a variety of *Bacillus* species and composed by glutamic acid monomers linked by amide bonds between α-amino and γ-carboxyl groups [10,11] (Figure 1). As PGA is nontoxic, and nonimmunogenic, it is successfully used in both biomedicine and food, cosmetics, agriculture, and wastewater industries [10,11]. PGA has also been widely used in drug release, because its carboxyl groups on the side chains represent effective attachment points for the conjugation and controlled delivery of therapeutic agents. Finally, PGA is known to act as a natural moisturizer and absorbent straight-chain macromolecule, its hygroscopic effect being comparable to that of hyaluronic acid [11].

PGA conformation was shown to be extremely flexible, depending on its concentration and pH of the solution. At low concentration (0.1% w/v) and at pH < 7.0, its structure is largely based on α-helices, whereas a β-sheet-based conformation, which allows the PGA to expose more of its negative charges, predominates at pH > 7.0 [12]. However, although it was demonstrated that PGA can give rise to fibers and membranes, it is unable to act as a thermoplastic polymer under ambient humidity [13]. Microscopic analyses of the PGA hydrogel revealed a multi-bag-like structure that enables it to absorb moisture five thousand times its own weight, the amount of water contained within the hydrogel being influenced by pH and salt content [14]. In fact, the degree of swelling was shown to increase with the parallel increase in both pH and the amount of ionized acidic side chains [15].

Therefore, as the usage of PGA as such to produce bioplastics has been so far unsuccessful [16], the present study was aimed to find out new experimental conditions (i.e., purification of the commercial product, FFS composition, and pH) for obtaining handleable PGA-based films with properties suitable for specific applications.

## 2. Materials and Methods

### 2.1. Materials

PGA, purchased from Xi’an Fengzu Biological Technology Co., Ltd. (Xi’an City, Shaanxi province, China), was dissolved in distilled water (0.4 g/mL) and, when required, extensively dialyzed against distilled water until a conductivity of 9 mS/cm was reached. Spectra/Por^®^3 dialysis membranes (standard regenerated cellulose tubing, 29 mm diameter; molecular weight cut-off 3.5 kDa) were from Sigma Chem. Co. (St. Louis, MO, USA). Glycerol (GLY, about 87%) was purchased from the Merck Chemical Company (Darmstadt, Germany). All other chemicals and solvents used in this study were analytical-grade commercial products.

### 2.2. Thermogravimetric Analisys (TGA)

TGA was performed with a Perkin-Elmer TGA6 thermobalance at a heating rate of 10 °C/min under a nitrogen atmosphere with a flow of 20 mL/min, in a temperature range of 30 to 500 °C. TGA experiments were carried out using 10 mg of PGA obtained by freeze-drying both undialyzed and dialyzed biopolymer solutions.

### 2.3. Zeta Potential Measurements

Zeta potential values were determined by a Zetasizer Nano-ZSP (Malvern®, Worcestershire, UK) equipped with an automatic titrator unit (Malvern® autotitrator mpt-2). The device was equipped with a helium–neon laser of 4 mW output power operating at the fixed wavelength of 633 nm (wavelength of laser red emission). The instrument software programmer calculated the zeta potential through the electrophoretic mobility by applying a voltage of 200 mV using the Henry equation. Water solutions of PGA (1.0 mg/mL), submitted or not to extensive dialysis, were brought to pH 12.0 by using 1.0 N NaOH; then, the titration was carried out from pH 12.0 to 2.0 by adding 1.0, 0.5, and 0.1 N HCl as titrant solutions under constant stirring at 25 °C. The zeta potential and mean hydrodynamic diameter (zeta-average) of the particles were measured, as previously described [17], at each pH value in triplicate.

### 2.4. Ultrafiltration and Nanofiltration Processes

**Figure 2:**
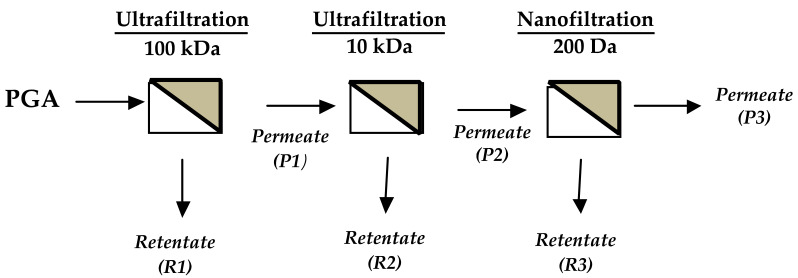
PGA downstream process flowchart.

Ultrafiltration processes were carried out using a polyethersulfone cassette membrane of 100 kDa and a 10 kDa cut-off, with a filtering area of 0.1 m^2^ (Sartorius Stedim, Gottingen, Germany). The system used for the membrane processes was a Sartoflow Alpha (Sartorius Stedim, Gottingen, Germany) equipped with a 10 L volume steel tank, with a jacket, pressure gauges on the inlet, retentate and permeate lines, and a thermostatic bath to keep the temperature constant. Undialyzed PGA (25 g) was dissolved in 2.5 L of deionized water, concentrated to 0.5 L on a 100 kDa cut-off membrane, and diafiltrated 2-fold with 1.0 L of deionized water. The obtained permeate (P1, 3.0 L) was concentrated to 0.25 L and 2-fold diafiltrated with deionized water on the 10 kDa cut-off membrane. The obtained permeate (P2, 3.25 L) was concentrated to 0.54 L on a nanofiltration membrane. The nanofiltration processes were performed using a polyethersulfone spiral membrane of 150–200 Da with a filtering area of 0.3 m^2^ (Fluxa Filtri, Milano, Italy). The collected retentates were collected and dissolved in distilled water to be analyzed. The PGA downstream process flowchart is illustrated in Figure 2.

### 2.5. Size-Exclusion Chromatography Coupled with Triple-Detection Analysis (SEC–TDA)

The chromatographic analyses of commercial PGA, as well as of the PGA fractions obtained by ultrafiltration and nanofiltration processes, were performed using a size-exclusion column coupled with triple-detection (laser light scattering, refractometry, and viscosity) equipment (Viscotek, Lab Service Analytica, Anzola dell’Emilia, Bologna, Italy). Homopolypeptide molecular weight distribution, molecular size distribution, polydispersity index, and intrinsic viscosity were derived as previously described [18]. The OmniSEC software program was used for the acquisition and analysis of the Viscotek data. Two TSK–GEL GMPWXL columns (Tosoh Bioscience, Rivoli, Torino, Italy, Cat. No. 8-08025, hydroxylated polymethacrylate base material, 100–1000 Å pore size, 13 μm mean particle size, 7.8 × 30.0 cm) in series that were preceded by a TSK–GEL guard column GMPWXL (Tosoh Bioscience, Cat. No. 08033, 12 μm mean particle size, 6.0 × 4.0 cm) were used. An isocratic elution with 0.1 M NaNO_3_ aqueous solution (pH 7.0) at a flow rate of 0.6 mL/min was carried out. Analyses were performed at 40 °C with a running time of 60 min. The dn/dc used for the analyses was 0.185 as found for protein [19].

### 2.6. ^1^H-NMR Spectroscopy

PGA was identified before and after dialysis of the commercial product, as well as after its ultrafiltration and nanofiltration processes, by ^1^H-NMR spectroscopy on a 400 Bruker Ascend and a 500 Varian Inova spectrometer (Bruker Corporation Billerica, MA, USA) operating at proton frequencies of 400 and 500 MHz. The solvent was D_2_O (HDO, δ 4.8).

### 2.7. Preparation and Casting of FFSs

Different FFSs (25 mL) containing 200–800 mg of either undialyzed or dialyzed PGA, as well as the retentates (R1, R2 and R3), obtained by undialyzed PGA ultrafiltration and nanofiltration processes, were prepared by adjusting the pH to different values (6.0, 5.0, 4.0, 3.5, 3.0, and 2.0) by 1.0 N HCl. All the FFSs were left stirring for 20 min and then, when required, different concentrations of GLY (1–50%, w/w PGA) were added. All the FFSs were poured onto polystyrene plates (1 mL × cm^2^), most experiments being carried out by using 8 cm diameter polystyrene Petri dishes, and were allowed to dry in an environmental chamber at 25 °C and 45% relative humidity for 48 h. Finally, when formed, the dried films were peeled from the casting surface and stored conditioned in an environmental chamber at 25 °C and 50% relative humidity for 2 h, by placing them into a desiccator over a saturated solution of Mg (NO_3_)_2_·6 H_2_O, before being analyzed.

### 2.8. Film Characterization

Tensile tests were carried out to evaluate film mechanical properties. An Instron model no. 5543A (Instron Engineering Corp., Norwood, MA, USA) electromechanical testing system was used for that purpose, and tensile strength (TS), elongation at break (EB), and Young’s modulus (YM) were measured on five specimens of each film sample (5 cm gage length, 1 kN load, and 1 mm/5 min speed). All the handleable films were cut into 1 cm × 8 cm strips by using a sharp scissor. One specimen of each sample (1 × 30 cm strip) was always prepared and analyzed with a specimen gage length of 25 cm [20] to confirm the data obtained with 1 × 8 cm film strips. Film thickness was measured in five different points with a micrometer (Electronic digital micrometer, DC-516, sensitivity 0.001 mm). Film transparency analysis was performed as described by Galus and Kadzinska [21] and the opacity was calculated as follows:

Opacity = A600nm/X(1)

where A600nm is the absorbance at 600 nm and X is the film thickness (mm).

Film surfaces were analyzed by scanning electron microscopy (SEM). Films were cut using scissors, mounted onto a stub, and sputter-coated with platinum–palladium (Denton Vacuum Desk V), before being observed by a Supra 40 Zeiss (Electron High Tension = 5.00 kV, in lens detector).

### 2.9. Statistical Analysis

JMP software 10.0 (SAS Institute, Cary, NC, USA), one-way ANOVA, and the least significant difference test for mean comparisons were used. Differences were considered to be significant at *p* < 0.05.

## 3. Results and Discussion

**Figure 3:**
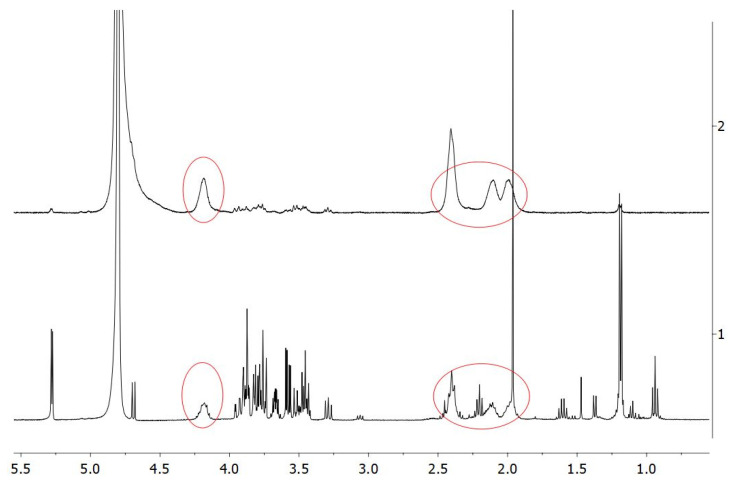
^1^H-NMR spectra of PGA before (lower panel) and after (upper panel) dialysis. The circled peaks indicate PGA signals [21], whereas the 4.8 ppm peak corresponds to HDO present in the solvent (D_2_O). Further experimental details are given in the text.

The purchased PGA, produced by bacterial fermentation, was preliminarily subjected to extensive dialysis to eliminate all the small-molecular-weight metabolites, mostly glucose, contained in the original homopolymer preparation and representing about 50% of the total dry weight. The ^1^H-NMR spectrum reported in the lower panel of Figure 3 clearly indicates the impurities present in the original PGA sample, impurities that were substantially absent after dialysis (Figure 3, upper panel). In fact, the circled peaks highlight the specific PGA signals [22] that are the main signals detected in the spectrum of the dialyzed PGA sample.

These results seem to be confirmed by TGA analyses of undialyzed and dialyzed samples. It is well known that the polymer thermal degradation is characterized by the decrease in its average molecular mass, as well as by the loss of its physical, mechanical, and electrical properties [23].

**Figure 4:**
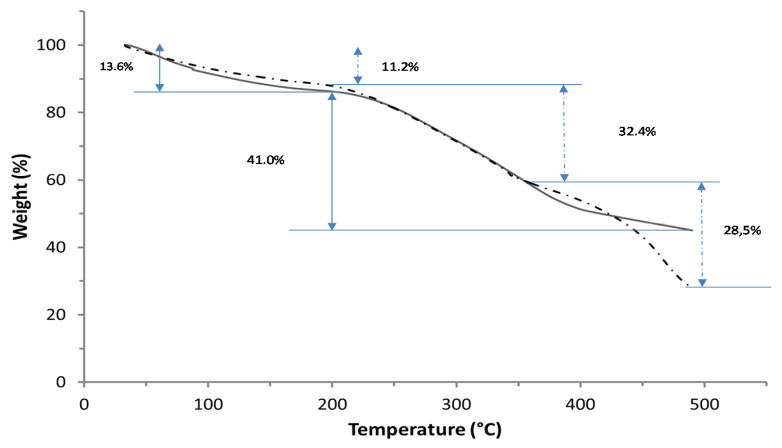
TGA curves of undialyzed (-·-·-) and dialyzed (**^____^**) PGA. Experimental details are given in the text.

TGA curves reported in Figure 4 revealed the first decomposition step in the 25–190 °C range, corresponding to a weight loss of 11.2% in the undialyzed PGA and of 13.6% in the dialyzed PGA, mainly as a consequence of the release of water absorbed by both samples due to their high hydrophilicity [24]. Moreover, a second decomposition step was evidentiated in the 210–400 °C range. The thermal PGA decomposition probably occurred through a mechanism of end-of-chain unzipping cyclodepolymerization releasing pyroglutamic acid units and, consequently, by a gradual decrease in the biopolymer length [24]. More in particular, the TGA curve corresponding to the undialyzed PGA sample showed a temperature interval (210–358 °C) shorter than that detected for the dialyzed biopolymer (210–490 °C) with a weight loss of 32.4% and 41.0% for the two samples, respectively. The larger temperature range of degradation of PGA present in the dialyzed sample indicates a higher thermostability of the biopolymer probably due to its higher crystallinity acquired following dialysis. In fact, the elimination of small-molecular-weight impurities might have triggered the aggregation of PGA chains. The additional step of decomposition (weight loss of 28.5%), observed by analyzing the undialyzed sample in the 434–500 °C range, would confirm such a hypothesis if associated with the thermal decomposition of the impurities.

**Figure 5:**
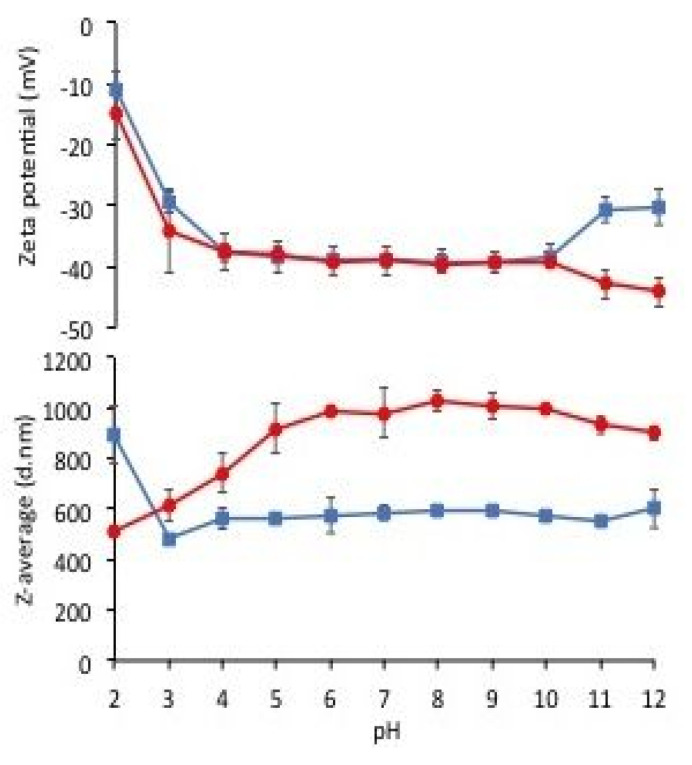
Zeta potential (upper panel) and Z-average (lower panel) measurements of PGA performed at different pH values. Undialyzed (■) and dialyzed (●) PGA samples containing 1.0 mg protein dissolved in 1.0 mL of distilled water adjusted at pH 12. All the results are reported as mean ± standard deviation. Further experimental details are given in the text.

Conversely, the zeta potential titration curves of PGA before and after PGA dialysis were superimposable, showing a progressive decrease in negative charge of the PGA particles under pH 4.0 (Figure 5, upper panel). In addition, the Z-average of the PGA particles was quite similar when measured between pH 3.0 and 4.0, i.e., at pH values where the biopolymer had its carboxyl groups only partially charged (Figure 5, lower panel). Therefore, these findings suggested preparing PGA FFSs at pH values between 3.0 and 10.0 in order to obtain stable solutions.

Preliminary attempts to produce films with the undialyzed biopolymer sample were unsuccessful. In fact, by casting and drying PGA FFSs prepared in the range of pH 3.0–10.0, both in the absence or presence of different concentrations of GLY (1–50% w/w PGA) added as a plasticizer, handleable films were not produced. This result could be explained with the possible negative interference by low-molecular-weight compounds, mostly glucose, present in the commercial PGA preparation, probably responsible for an excessive plasticizer effect hindering the production of manipulatable material.

Conversely, by casting FFSs containing dialyzed PGA alone, yellowish handleable films were produced in the absence of plasticizer only in the range of pH between 3.0 and 4.0. Sticky and nonpeelable materials were obtained at pH values lower than 3.0 and higher than 4.0, probably as a consequence of the unfavorable dissociation of PGA carboxyl groups. Moreover, the analysis of the mechanical properties of the obtained materials indicate a marked decrease in both TS and YM, with a concomitant increase in film EB and opacity, by enhancing the pH of the FFS from pH 3.0 to 3.5 and 4.0 (Table 1).

Therefore, further experiments were carried out to investigate the effect of increasing concentrations of GLY added to the dialyzed PGA FFSs prepared at pH 3.5. Marked enhancements in EB of the produced films were observed when very low amounts (1–4%, w/w of PGA) of plasticizer were present in the FFSs, while very sticky and unhandleable materials were produced at GLY concentrations higher than 4%. The obtained PGA films became much more extensible, maintaining their resistance and plasticity, as shown by the slight TS increase and the unmodified YM detected by analyzing the films containing 1, 2, and 4% GLY (Table 2).

The awesome film extensibility is clearly shown in the Instron analysis images inserted in Table 2, being irrefutable proof of the special behavior of GLY-plasticized PGA material.

**Figure 6:**
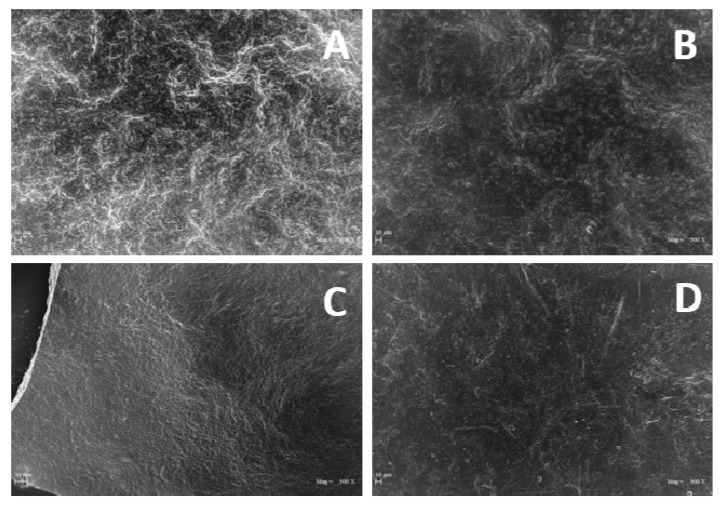
SEM micrographs of the surfaces (500× magnification) of films obtained with dialyzed PGA (800 mg) at pH 3.5 in the absence (**A**) and presence of 1% (**B**), 2% (**C**), and 4% (**D**) GLY. The images shown were chosen as the most representative of each sample. Further experimental details are given in the text.

SEM images reported in Figure 6 show the surfaces of the GLY-containing films smoother than that of the film prepared in the absence of plasticizer, even though no significant differences were observed among the films obtained at different GLY concentrations.

In order to characterize the PGA molecular species contained in the FFSs, giving rise to the analyzed films, the undialyzed homopolypeptide was subjected to membrane ultrafiltration and nanofiltration processes, and, subsequently, SEC-TDA analyses of the obtained retentate fractions R1, R2, and R3 (derived from 100 kDa, 10 kDa, and 200 Da cut-off membranes, respectively) were carried out, as well as of the undialyzed PGA sample before processing.

Table 3 resumes the main parameters of the ultrafiltration and nanofiltration experiments and the % of PGA recovered with respect to the undialyzed PGA processed, whereas Table 4 reports the molecular weight, the polydispersity index, the intrinsic viscosity, and the representativity of the samples.

**Figure 7:**
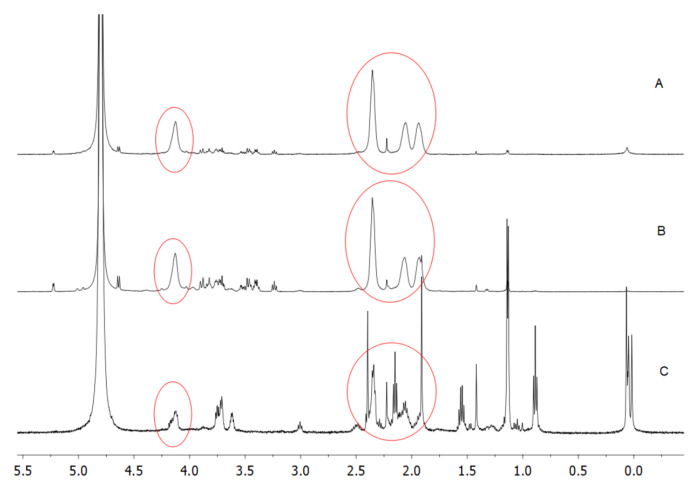
^1^H-NMR spectra of PGA contained in R1 (**A**), R2 (**B**), and R3 (**C**). The circled peaks indicate the homopolypeptide signal [22], whereas the 4.8 ppm peak corresponds to HDO present in the solvent (D_2_O). Further experimental details are given in the text.

These data indicate the presence of two peaks in R1, the first corresponding to a biopolymer of about 120 kDa with a representativity of 33.5% and the second one to a biopolymer of about 30 kDa with a representativity of 63.6%, while only one biopolymer peak of about 18.5 kDa, with a representativity of 91%, was revealed in R2. ^1^H-NMR spectra shown in Figure 7 confirmed the presence of PGA in R1 (A), R2 (B), and R3 (C) samples.

Finally, FFSs prepared at pH 3.5 with the three samples were casted and dried as reported in the Materials and Methods, and the obtained results showed that R1 and R2 were able to give rise to handleable films, whereas R3 produced only a very sticky and nonpeelable material. Furthermore, the results reported in Table 5 show marked differences between the properties of the films derived from R1 and R2 samples, the opacity, TS, and YM being much higher than those in R1, which contained PGA with higher molecular weights, whereas both EB values were found lower than those detected with R2, in which a PGA molecular form with lower mol. wt. was present. These results are in strict agreement with the following equation, indicating that polymer tensile strength rises with the increase in its molecular weight:

*σ* = *σ*∞ − A/M *σ*∞ (2)

where *σ*∞ is the tensile strength of the polymer with a molecular weight of infinity, A is some constant, and M is the molecular weight [25]. Therefore, the polymer chains with a lower molecular weight are weakly bonded and can move easily, whereas, in the case of larger-molecular-weight polymers, their chains are entangled, giving high strength to the polymer.

**Figure 8:**
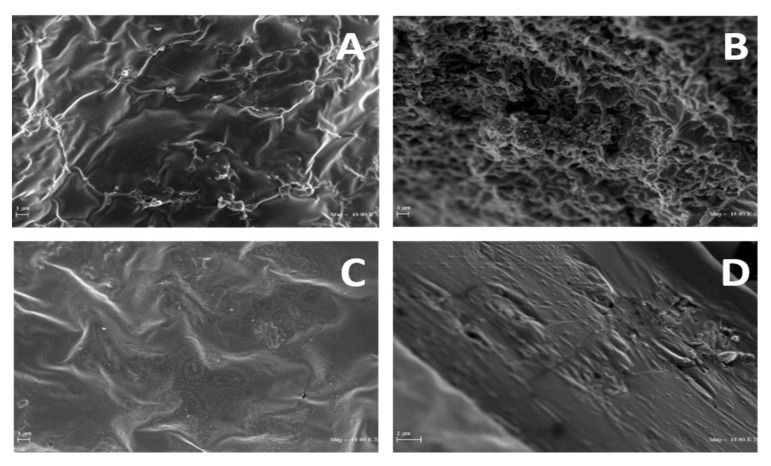
SEM micrographs of PGA films prepared with R1 (**A**,**B**) and R2 (**C**,**D**) samples (800 mg) at pH 3.5. The images of the film surfaces (**A**,**C**) and cross-sections (**B**,**D**) shown were chosen as the most representative of each sample (10,000× magnification). Further experimental details are given in the text.

In addition, SEM micrographs reported in Figure 8 show evident differences in both surfaces and cross-sections among PGA films obtained from R1 and R2 samples, the R1-derived films appearing more irregular and rough than those prepared with R2, which exhibited a smoother surface and a more homogeneous cross-section.

All these data strongly suggest that PGA chain length is responsible not only for the capacity of the homopolymer to film or not, the very-low-molecular-weight PGA being unable to give rise to any manipulatable material, but also for conferring to the films different features depending on homopolymer size.

## 4. Conclusions

Plastic materials were obtained from an inexpensive commercial source of PGA that was preliminarily subjected to extensive dialysis or ultrafiltration and characterized. Handleable and extensible films were produced only at low values of pH (between 3 and 4) and without plasticizer addition. However, the presence of very low concentrations of glycerol markedly increased the plastic features of the prepared material. The ultrafiltration fractions containing different molecular species of the homopolypeptide gave rise to films exhibiting completely different mechanical properties among them, suggesting that PGA chain length is responsible for conferring to the films different features depending on homopolymer size. PGA represents, thus, a potential candidate of bio-based homopolymer to produce bioplastics with different characteristics, which is useful for a variety of applications depending on the length of its chain. The low cost of raw PGA preparation (1–10 USD/kg), being in the same cost range of the oil-derived plastic polymers (1.2–1.8 USD/kg) [26] and of some other commercially available biopolymers, such as polylactic acid, confirms the prospective use of such a homopolymer to produce materials alternative to the conventional plastics.

## Figures and Tables

**Table 1:** Images and properties of films obtained at different pH values (A, pH 3.0; B, pH 3.5; C, pH 4.0) with 800 mg of dialyzed homopolypeptide preparation. Experimental details are given in the text.

	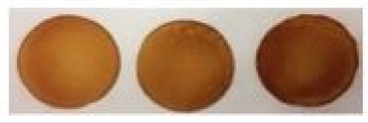		
Film Property *	A	B	C
Thickness (µm)	80 ± 5	81 ± 3	111 ± 16
Opacity (mm^−1^)	9.1 ± 0.2	10.1 ± 0.6	15.5 ± 1.1
TS (MPa)	1.8 ± 0.3	0.2 ± 0.1	0.1 ± 0.1
EB (%)	14 ± 9	450 ± 32	268 ± 25
YM (MPa)	122 ± 11	3 ± 1	6 ± 1

* TS, tensile strength; EB, elongation at break; YM, Young’s modulus.

**Table 2:** Images and properties of the films obtained at pH 3.5 with 800 mg of dialyzed PGA preparation in the presence of different glycerol (GLY) concentrations. Images of tensile tests, before and after film stress, are also reported. Further experimental details are given in the text.

	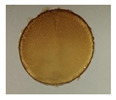	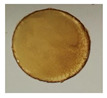	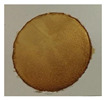	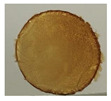
Film Property *	−GLY	+1% GLY	+2% GLY	+4% GLY
Thickness (µm)	80.0 ± 1.0	82.3 ± 2.1	85.0 ± 1.4	86.1 ± 2.8
Opacity (mm^−1^)	9.2 ± 0.1	9.5 ± 0.3	9.3 ± 0.1	8.1 ± 0.2
TS (MPa)	0.2 ± 0.1	0.2 ± 0.1	0.3 ± 0.1	0.4 ± 0.1
EB (%)	424 ± 20	580 ± 13	805 ± 8	969 ± 14
YM (MPa)	3 ± 1	3 ± 1	3 ± 1	3 ± 1
	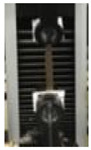	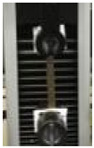	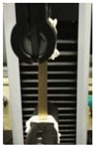	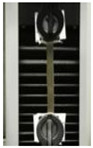
	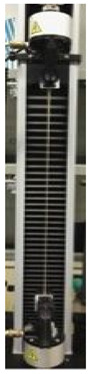	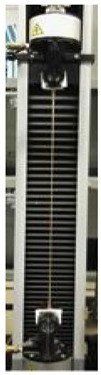	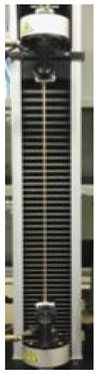	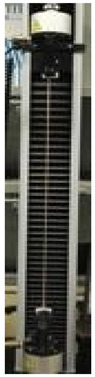

* TS, tensile strength; EB, elongation at break; YM, Young’s modulus.

**Table 3:** Ultrafiltration (UF) and nanofiltration (NF) process parameters and PGA recovered with respect to the undialyzed PGA processed. Flux is the volume permeated over time and TMP is the transmembrane pressure.

Process	Flux (L/m^2^ · h)	TMP (bar)	PGA Recovered (%)
UF (100 kDa, R1)	120	0.25	25.2
UF (10 kDa, R2)	12	0.30	23.0
NF (150-200 Da, R3)	60	8.2	43.6

**Table 4:** SEC–TD characterization of the samples following concentration and diafiltration of the undialyzed PGA on 100 kDa membranes (R1) and of the retentate obtained after concentration and diafiltration of the deriving permeate on 10 kDa membranes (R2). Further experimental details are given in the text.

Sample	dn/dc	Ret. Vol. (mL)	Mw (kDa)	Mw/Mn	IV (dL/g)	Representativity (%)
Undialyzed PGA	0.185	16.82	43.42	2.04	0.69	93.0
R1	0.185	15.95	119.59	1.59	1.51	33.5
	0.185	16.41	30.68	1.13	0.503	63.6
R2	0.185	17.01	18.42	1.66	0.422	90.7

**Table 5:** Images and properties of films obtained at pH 3.5 with 800 mg of PGA contained in R1 and R2 samples. Experimental details are given in the text.

	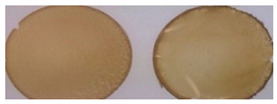	
Film Property	R1-PGA	R2-PGA
Thickness (µm)	75 ± 6	62 ± 3
Opacity (mm^−1^)	18.9 ± 1.2	9.9 ± 1.4
TS (MPa)	12 ± 2	2 ± 1
EB (%)	0.9 ± 0.2	47.7 ± 9.8
YM (MPa)	1962 ± 222	138 ± 10
